# Swelling, Mechanics, and Thermal/Chemical Stability of Hydrogels Containing Phenylboronic Acid Side Chains

**DOI:** 10.3390/gels4010004

**Published:** 2017-12-29

**Authors:** Arum Kim, Heelim Lee, Clinton F. Jones, Siddharthya K. Mujumdar, Yuandong Gu, Ronald A. Siegel

**Affiliations:** 1 Department of Biomedical Engineering, University of Minnesota, Minneapolis, MN 55455, USA; arumkim@vaumc.org (A.K.); siddharthya.mujumdar@roche.com (S.K.M.); 2 Department of Chemical Engineering and Materials Science, University of Minnesota, Minneapolis, MN 55455, USA; xilin2@snu.ac.kr; 3 Department of Pharmaceutics, University of Minnesota, Minneapolis, MN 55455, USA; clinton.f.jones@gsk.com (C.F.J.); guyd@ime.a-star.edu.sg (Y.G.)

**Keywords:** gels, sugar-sensitive, equilibria, mechanical relaxation, dynamic crosslinks, thermal/chemical degradation

## Abstract

We report here studies of swelling, mechanics, and thermal stability of hydrogels consisting of 20 mol % methacrylamidophenylboronic acid (MPBA) and 80 mol % acrylamide (AAm), lightly crosslinked with methylenebisacrylamide (Bis). Swelling was measured in solutions of fixed ionic strength, but with varying pH values and fructose concentrations. Mechanics was studied by compression and hold. In the absence of sugar or in the presence of fructose, the modulus was mostly maintained during the hold period, while a significant stress relaxation was seen in the presence of glucose, consistent with reversible, dynamic crosslinks provided by glucose, but not fructose. Thermal stability was determined by incubating hydrogels at pH 7.4 at room temperature, and 37, 50, and 65 °C, and monitoring swelling. In PBS (phosphate buffered saline) solutions containing 9 mM fructose, swelling remained essentially complete for 50 days at room temperature, but decreased substantially with time at the higher temperatures, with accelerated reduction of swelling with increasing temperature. Controls indicated that over long time periods, both the MPBA and AAm units were experiencing conversion to different species.

## 1. Introduction

Phenylboronic acids (PBAs) are compounds that bind to carbohydrates and other polyols by reversible bidentate condensation [1,2,3]. Hydrogels containing PBA sidechains have been studied and used for chromatographic separations and cell sorting [4,5,6], as components of sensors for glucose and other sugars [7,8,9,10,11,12,13,14,15,16,17,18,19,20,21,22,23,24,25,26,27,28,29,30], and as components of drug delivery systems capable of releasing insulin when triggered by the presence of glucose [26,31,32,33,34,35]. The last two applications have received much interest in the past two decades, since they represent an attractive alternative to enzyme-mediated glucose sensors and drug delivery devices. Enzyme-based sensors are subject to fouling and denaturation over time, and when coupled with hydrogel swelling, they rely on the production of acidic protons, which are rapidly buffered in the physiological host medium. Hydrogel sensors based on PBAs respond by mass action kinetics, and are not susceptible to the degradation processes that occur with enzymes.

**Scheme 1:**
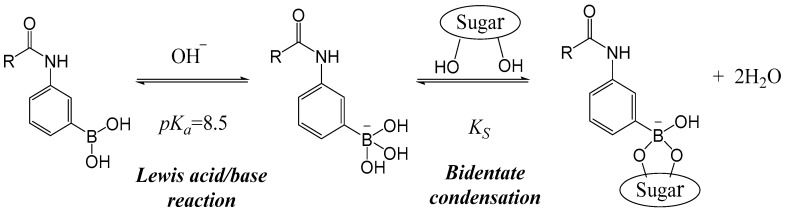
Reactions of amido-phenylboronic acids (PBAs) with hydroxide ion and sugars. The symbol *K_S_* refers to the dissociation constant associated with the bidentate condensation reaction. The *pK_a_* value is suggested by the literature [7]. R denotes the polymer backbone. Reproduced from Ref. [37].

The swelling mechanism of hydrogels containing PBA sidechains has been discussed many times in the literature [8,21,28,33,36,37]; we offer a brief synopsis, aided by Scheme 1, which is specialized to amido-PBAs. At relatively low pH, the boron atom of PBA is bonded to the phenyl ring and two –OH groups in a trigonal configuration. The boron’s valence shell contains an empty orbital, enabling Lewis acid–base pairing with :OH^−^. The result is a negatively charged boronate, with bonds emanating from the boron in a tetragonal configuration. This form is amenable to reversible bidentate condensation with properly configured –OH pairs on sugar molecules, stabilizing the ionized form. An increase in pH and/or sugar concentration increases the charge on the PBA moiety, promoting swelling due to the Donnan effect. Scheme 1 leaves out direct bidentate condensation of sugar to the uncharged trigonal boronic acid, which is much weaker, and hence much less populated than the ionized forms, at least for methacrylamidophenylboronic acid (MPBA) [7,38], which is the focus of this paper.

**Scheme 2:**
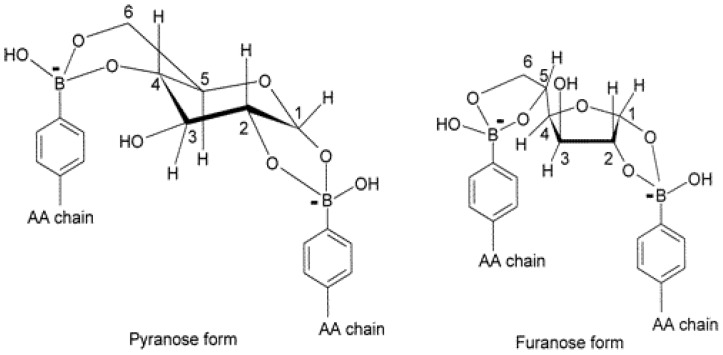
Putative structures of bis-bidentate crosslinking complexes between two PBA units on separate polymer chains, which are formed by glucose. Reproduced from Ref. [8].

This mechanism will apply to other sugars and molecules having properly configured –OH groups, so the mechanism is not specific to glucose [7,21]. For example, MPBA exhibits approximately 40 times greater binding affinity (*K*_S_) to fructose compared to glucose [8]. However, the concentration of glucose in human plasma is exceeds that of fructose by >200-fold, so fructose probably will interfere negligibly in glucose sensing and glucose triggered drug delivery applications. Nevertheless, fructose is a useful probe for binding studies. Fructose can only form a monobidentate condensation complex with PBA. Glucose, in either its pyranose or its furanose form, can form bis-bidentate complexes with two ionized PBA units at once, increasing the apparent crosslink density of the hydrogel, as depicted in Scheme 2 [8,35,39]. Under suitable conditions, this leads to shrinking of the hydrogel in the presence of glucose.

The present paper provides several contributions to the understanding of hydrogels based on PBAs. We study swelling, mechanics, and thermal stability of hydrogels containing 20 mol % MPBA and 80 mol % acrylamide (AAm) (p(AAM-*co*-MPBA)), which is a popular formulation, since MPBA groups are intrinsically hydrophobic, and the AAm comonomer endows the crosslinked polymer network with adequate swelling and solute permeability.

In the first set of studies, we analyze data from a previously report, in which we systematically varied pH and sugar (fructose or glucose) concentrations and measured the degree of swelling [37]. Addition of fructose leads to a parallel shift in the pH-swelling isotherm, while addition of glucose leads to more complex behavior, due to the crosslinking that occurs according to Scheme 2. We also fit pH/fructose swelling isotherms to Flory–Rehner–Donnan–Langmuir (FRDL) theory [7,40], augmented to include fructose binding to the hydrogel.

In a second set of studies, we demonstrate the transient nature of the glucose-mediated crosslinks by mechanical measurements. It is shown that following rapid compression, the modulus of MPBA is initially high, but eventually relaxes to a plateau value. This relaxation is attributed to rearrangement of glucose-mediated crosslinks. We also measure the modulus in the absence of sugar or in the presence of fructose as a check on the estimated crosslink density inferred from the FRDL fits.

In the third set of studies, we expose the MPBA-*co*-AAm hydrogels, at pH 7.4, to fructose for long periods of time at several temperatures. By following swelling as a function of time, we determine the rate of degradation of the boronic acid moieties. These studies appear to be the first of their kind with MPBA-*co*-AAm hydrogels, and the results may be important for those who wish to use such hydrogels for long term applications.

## 2. Results

### 2.1. Equilibrium Swelling

**Figure 1:**
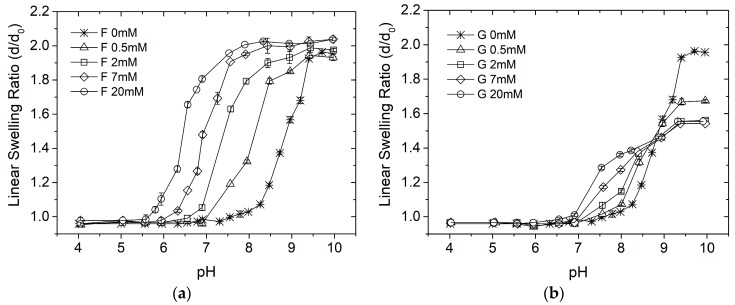
Equilibrium swelling of p(AAM-*co*-MPBA) hydrogels in buffers of various pH, and at varying concentrations of (**a**) fructose (F); and (**b**) glucose (G). All measurements were performed in duplicate. Replotted from Ref. [37].

Equilibrium swelling data, taken from Ref. [37] in hydrogels prepared as described above, are shown for fructose and glucose in Figure 1a,b, respectively. Data collected in sugar-free solutions is included in both plots. Data are reported as $d/d_{0}$, the ratio of equilibrium diameter at a given condition to the diameter of the gel at synthesis (in the capillary). The swelling curves are sigmoidal, as expected for pH-sensitive hydrogels. The curves at different fructose concentrations are nearly exact translates along the pH scale, which is consistent with Scheme 1, assuming that degree of ionization is the sole determinant of swelling. Actually, the data indicate that fructose binding causes a slight increase in swelling. This effect will be ignored here, in order to keep the analysis and the modeling simple. Before going into detailed modeling, we may note that according to Scheme 1, the apparent acidity constant, *pK_a_*, of the MPBA moiety will be altered from its value in the absence of sugar, pK_0_, according to

(1) $$pK_{a}=pK_{0}-\log_{10}\left(1+\frac{C_{S}}{K_{S}}\right)$$

where is the sugar’s dissociation constant from ionized, tetragonal MPBA (Scheme 1), and is the fructose concentration. The acid shifts in the data at different fructose concentrations are well accounted for by assuming = 0.09 mM. This value is lower than the literature value for binding of fructose to free ionized PBA, 0.27 mM [1]. Apparently, fructose binding to MPBA is promoted in the hydrogel environment.

The swelling isotherms in the presence of glucose do not behave as simply as those for fructose. Below pH ≈ 8.8, increasing glucose concentrations leads to greater swelling, but not as simple translations of the sugar-free isotherm. The swelling curves crossover near pH 8.8, and above that pH, increased glucose concentration leads to relative shrinkage of the hydrogel. These observations are consistent with formation of crosslinks via bis-bidentate binding of glucose to MPBAs on separate chains.

A quantitative accounting for swelling as a function of pH and fructose concentration is provided by the FRDL model of pH-sensitive hydrogel swelling, augmented by a provision for fructose binding to the ionized form of MPBA [7,40]. Letting $\varphi_{0}$ and φ denote, respectively, the volume fraction of polymer in the hydrogel at synthesis and at swelling equilibrium, we have $\varphi /\varphi_{0}={\left(d/d_{0}\right)}^{-3}$. Taking into account the polymer/water mixing, polymer elasticity, and ion osmotic contributions to the swelling free energy, the equilibrium swelling condition is

(2) $$\ln(1-\varphi )+\varphi +\chi \varphi^{2}+{\bar{v}}_{w}\rho_{0}\left[{\left(\varphi /\varphi_{0}\right)}^{1/3}-(\varphi /2\varphi_{0})\right]-{\bar{v}}_{w}C_{salt}(\lambda +1/\lambda -2)=0$$

subject to the condition of electroneutrality inside the hydrogel,

(3) $$(1-\varphi )(\lambda -1/\lambda )C_{salt}-f\sigma_{0}(\varphi /\varphi_{0})=0$$

In these expressions, χ is the Flory–Huggins interaction parameter, $\rho_{0}$ and $\sigma_{0}$ are the molar densities of elastic chains and ionizable MPBA groups in the hydrogel at formation, respectively, while ${\bar{v}}_{w}$ and $C_{salt}$ are, respectively, the molar volume of water and the molar concentration of salt (NaCl + buffer) in the environment bathing the hydrogel. The terms λ and *f* represent, respectively, the Donnan ratio, describing partitioning of salt ions between the external solution and the hydrogel, and the fraction of ionizable groups that are ionized. The model is completed by specifying *f* according to

(4) $$f=\frac{1}{1+\lambda 10^{-(pH-pKa)}/(1+C_{S}/K_{S})}.$$

**Figure 2:**
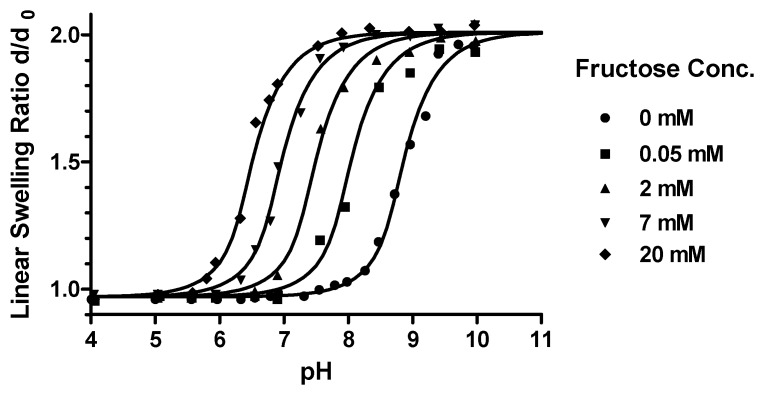
Fit of Flory–Huggins–Donnan–Langmuir (FRDL) model Equations (3)–(4) to fructose data in Figure 1a.

Figure 2 shows nonlinear least squares fits of the model to the data of Figure 1. In fitting the model using Matlab (Mathworks: nlinfit and nlparci functions), the initial volume fraction and charge density were calculated from the feed composition, assuming essential completeness of the gelation reaction. The initial molar density of active chains, was not fixed, however, since degree of incorporation of crosslinker (Bis), is known to not correlate reliably with the number of elastically active chains, due to the presence of dangling ends and loops. The model fits very well, and the parameter estimates, shown with their 95% confidence intervals in Figure 2, are tight. As will be seen below, the estimated value of $\rho_{0}$ is consistent with independent mechanical measurements. It is apparent that the model can be used to reliably predict swelling under any typical pH and fructose concentration conditions, at least at room temperature. The χ parameter, in particular, may change with temperature.

The fitted value of χ is higher than that used (~0.5) by other researchers who have studied MPBA-*co*-AAM hydrogels with much lower PBA content, e.g., [7]. The higher value signals the hydrophobic quality that is imparted by the relatively large mole fraction of MPBA.

The fitted acidity constant, $pK_{0}$ = 8.20 is substantially lower than 8.86, the value that has been measured for PBA, and lower than 8.5, measured for amido-PBA [7]. Within the present model, the fitted value is difficult to doubt, however, since changes would lead to a lateral shift in the predictions. However, there may be other causes of the acid shift in the swelling curves. For example, the overall polarity of the MPBA moiety may increase with ionization, and there may be significant changes in ion-dipole, van der Waals, hydrogen bonding, and/or hydrophobic interactions when MPBA is ionized. To investigate this aspect further, we considered a model variant in which the χ parameter decreases with increasing ionization. This variant shifted the apparent $pK_{0}$ of MPBA to higher values, but the whole range of data was fitted only when the estimated value of ρ_0_ increased beyond the range that is consistent with the mechanical data to be presented below. For this reason, we retain the present model with the unexpectedly low value of $pK_{0}$.

### 2.2. Mechanics

As indicated above, p(AAM-*co*-MPBA) hydrogels swell either due to increase in pH or increase in fructose concentration. Glucose, on the other hand, causes the hydrogels to shrink at high pH, and it is believed that this behavior is due to formation of bis-bidentate crosslinks (Scheme 2), which does not occur with fructose. Since the bidentate binding of sugars to MPBA is reversible, it follows that the glucose-mediated crosslinks must be in a state of dynamic equilibrium. Here, we probe the dynamic reorganization of glucose-mediated crosslinks by mechanical compression. We also use mechanical compression to provide an independent estimate of the number density of active chains, $\rho_{0}$.

**Figure 3:**
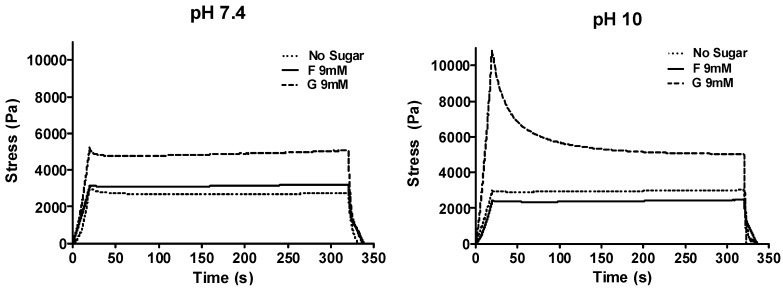
Engineering stress records for p(AAM-*co*-MPBA) hydrogels following compression to 90% of original thickness. F and G denote fructose and glucose, respectively.

Figure 3 shows the results of compress–hold–decompress experiments for hydrogels that were pre-equilibrated at pH 7.4 or 10.0, in sugar-free solutions, or in the presence of 9 mM fructose or glucose. At pH 7.4, the engineering stress, $F(t)/A_{i}$, reached a constant plateau, with negligible relaxation. At pH 10.0, hydrogels exposed to sugar-free and fructose-containing solutions exhibited the same behavior. However, at pH 10 with 9 mM glucose, the stress in the hydrogel exhibited a sharp peak at the end of the compression phase and then relaxed, over minutes, to a substantially smaller plateau, about 50% of the early peak.

According to the theory of rubber elasticity, extended to hydrogels [41], the plateau modulus is related to the molar density of active polymer chains, $\rho_{0}$, at hydrogel formation by the formula

(5) $$G_{\infty }=\frac{F_{\infty }/A_{0}}{\left(\alpha -\alpha^{-2}\right)}=RT\rho_{0}{\left(\varphi /\varphi_{0}\right)}^{1/3}=RT\rho_{0}(d_{0}/d),$$

where α is the compression ratio, $d/d_{0}$ is the linear swelling ratio of the hydrogel, and $F_{\infty }/A_{i}$ is the plateau engineering stress. Table 1 shows calculated values of $\rho_{0}$ based on the data of Figure 3. Values of $\rho_{0}$ for all but the hydrogel exposed to glucose at pH 10 cluster near 7 mM. This average value is very close to the value 7.26 mM estimated for $\rho_{0}$ based on the constant χ model presented in the previous section. The larger values of $\rho_{0}$ measured with glucose, especially at pH 10, suggest that dynamic crosslinks may contribute to the plateau modulus. (We note, however, that the somewhat lower value of $\rho_{0}$ for 9 mM fructose at pH 10 signals the possibility that the higher values observed with glucose may be due to statistical variation.)

**Figure 4:**
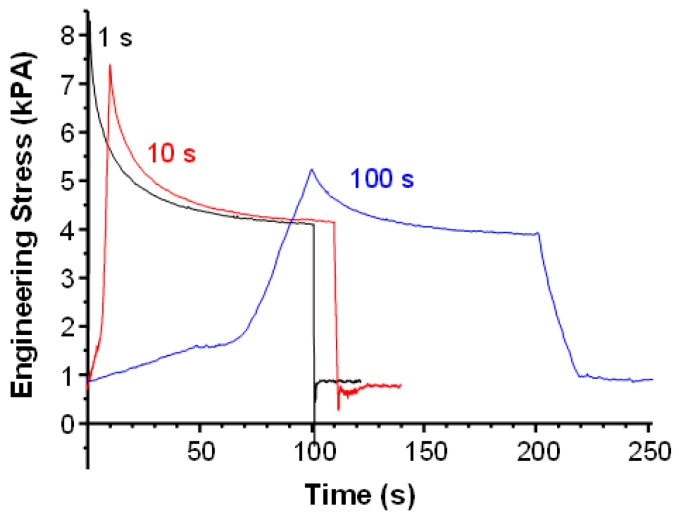
Mechanical compression engineering stress records for MPBA-*co*-AAm hydrogels in 9 mM glucose solutions at pH 7.4, with compression and decompression occurring of 1 s (black curve), 10 s (red curve), and 100 s (blue curve). The hold phase was 100 s in all cases.

To further study relaxations in the presence of glucose, faster and slower compression phases were applied at pH 10.0 and 9 mM glucose. Results, all gathered using the same hydrogel, are shown in Figure 4. In these experiments, there was an initial “soft” phase, during which the hydrogel, which may have been slightly curved, flattened between the two plates. With slower compression, the peak engineering stress was reduced, but all hydrogels relaxed to essentially the same plateau. This figure suggests that crosslink exchange commences during the compression phase. The slower the compression, the more equilibrated the crosslinks are at each stage.

The mechanical relaxations in the presence of glucose occur over minutes, and are not well fitted by a single decaying exponential. Very good fits were achieved using a variant of the Kohlrausch–Williams–Watts (KWW) stretched exponential form for the stress transient during the hold phase, $\sigma (t-T_{comp})$, i.e.,

(6) $$\sigma (t-T_{comp})=\sigma_{\infty }+\left[\sigma (T_{comp})-\sigma_{\infty }\right]e^{{\left[(t-T_{comp})/\tau \right]}^{\beta }},$$

where $\sigma (T_{comp})$ is the peak stress obtained at the end of the compression phase, occurring at $t=T_{comp}$, $\sigma_{\infty }$ is the plateau stress, τ is a time constant, and β is the stretch parameter. Table 2 displays values of τ and β for the three tested compression times, $T_{comp}$. Both τ and β show an increasing trend with compression time, while peak stress decreases.

The KWW form is often assumed to reflect a broad spectrum of relaxations, which are observed in glasses [42], albeit with much longer average relaxation times. In the present case, we suppose that processes of reversible bis-bidentate complex breaking and reformation, and chain undulation, contribute to this spectrum. Following application of strain, an instantaneous network consisting of permanent and transient crosslinks will be present, giving rise to the peak stress. As time progresses, crosslink rearrangements enable topological rearrangement of chains with respect to the transient crosslinks, with relaxation of stress. The stretched exponential form may reflect topological rearrangements over multiple length scales.

**Figure 5:**
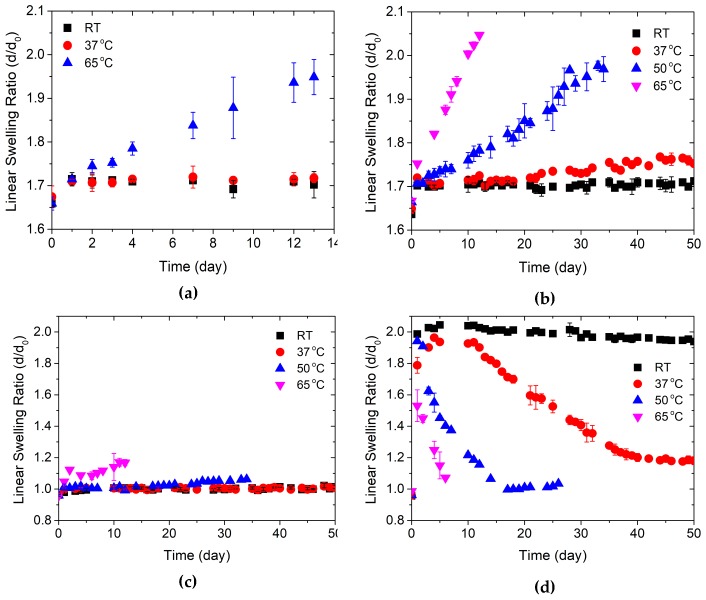
Swelling and shrinking kinetics at various temperatures in (**a**) pAAm hydrogels incubated in phosphate buffered saline (PBS); (**b**) pAAM hydrogels incubated in PBS with 9 mM fructose; (**c**) p(AAM-*co*-MPBA) hydrogels incubated in PBS; and (**d**) p(AAM-*co*-MPBA) hydrogels incubated in PBS with 9 mM fructose. In all cases, pH = 7.4 and ionic strength = 0.155 mM. All measurements performed in duplicate. RT denotes room temperature (25 °C).

With slower compression, it is expected that the faster relaxations will be complete before the hold phase commences. In other words, slow compression represents a kind of low-pass filtering of the relaxation spectrum. This explains the trend towards increase in “average” relaxation time, τ. The stretch exponent β, which represents the heterogeneity of relaxation times, approaches unity as the relaxation spectrum narrows. It is interesting to note, in this regard, that if one fits the $T_{comp}$ = 1 s data in Figure 5, but only considers data taken after 10 s of hold, then the values τ = 17.8 ± 0.3 and β = 0.933 ± 0.023 are obtained. 

Further experimental and theoretical work probing relaxations in the presence of glucose may be a fertile area for future research. It has been shown that the rates of condensation and hydrolysis of glucose with PBA are strongly dependent on temperature, even though the equilibrium constant is not so strongly affected [43]. Temperature may, therefore, have a strong effect on relaxation kinetics.

### 2.3. Thermal Stability

Phenylboronic acids are known to undergo degradation, while acrylamide units hydrolyze to acrylate ion in alkaline environments. If p(AAM-*co*-MPBA) hydrogels are to be used in long-term implants, then their chemical stability needs to be ensured. In the following, we present experiments in which swelling of both pAAm and p(AAM-*co*-MPBA) hydrogels was monitored for up to 60 days, at various temperatures, in either sugar-free or 9 mM fructose-containing PBS buffers at pH 7.4 and ionic strength 0.155 mM.

Results are shown in Figure 5a–d. In all cases, hydrogel cylinders were exposed to the medium, and swelled to an initial quasi-equilibrium swelling state, before the chemical degradation processes started to take over. Initial linear swelling ratios (*d*/*d*_0_) were near 1.7 for the pAAm hydrogels (Figure 5a,b), regardless of presence or absence of fructose. For the p(AAM-*co*-MPBA) hydrogels, initial swelling was reduced relative to the AAm hydrogels in sugar-free PBS, due to the hydrophobicity of the MBPA units (Figure 5c), but was substantially increased compared to AAm hydrogels in the presence of fructose, due to ionization of the MPBA (Figure 5d).

Figure 5a,b show the effects of temperature on long-term swelling kinetics of pAAm hydrogels in sugar-free PBS and in PBS with 9 mM fructose, respectively. In both cases, swelling was essentially constant at room temperature and at 37 °C up to at least two weeks. A slight increase in swelling was observed after about 20 days in fructose solutions at 37 °C. (Measurements were not made beyond 14 days in the absence of fructose.) Increasing temperature to 50 and 65 °C, swelling increased substantially, with the presence of fructose again displaying a small, but measurable effect at 65 °C. The influence of fructose on swelling in these hydrolyzing pAAm hydrogels is not understood at present.

Figure 5c displays measurements for the p(AAM-*co*-MPBA) hydrogels in the absence of fructose. In pure PBS, swelling was minimal and essentially constant at room and body temperatures. Swelling increased slowly at 50 °C. At 65 °C, there was a significant increase in swelling with time. Swelling was always lower than in Figure 5a, due to the hydrophobicity of the MPBA comonomer.

The data in Figure 5d shows that there were substantial chemical changes in the p(AAM-*co*-MPBA) hydrogels over time, above room temperature. At room temperature, swelling was mostly stable for the whole run of the experiment, although there was a slow but detectable decrease in swelling over time. At body temperature, swelling decreased monotonically from days 10–40, and then leveled off. This shrinkage following initial swelling was accelerated progressively at 50 and 65 °C.

The cause of this deterioration of swelling in p(AAM-*co*-MPBA) hydrogels was almost certainly degradation of MPBA, with disruption of the mechanism illustrated in Scheme 1. The precise mechanism of degradation cannot be determined by our data, since we did not chemically analyze the hydrogel or its degradation products. However, it is known that the B–O bond is more stable than the B–C bond, and that arylboronic acids are susceptible to oxidation by O_2_ [44]. Buffers were refreshed frequently, providing a renewed if not well-controlled source of O_2_. A likely degradation product, then, was boric acid, leaving behind a nonionizable benzyl acrylamide (BzA) functionality on the hydrogel, which caused the hydrogel to shrink. In this regard, it is noteworthy that the hydrogels initially containing MPBA shrink below the swelling degrees achieved at the same temperatures in the AAm homopolymer hydrogels. Further studies in deoxygenated buffers, or buffers prepared with varying pH and pO_2_ values, in which degradation products are analyzed, could confirm the role of oxygen in the degradation of the hydrogels’ response to fructose. Monitoring oxygen consumption during hydrogel degradation in a closed batch reactor could provide information regarding kinetics of degradation.

While degradation of the MPBA unit is of most interest, it should be pointed out that the time course of swelling is influenced by both putative MPBA oxidation and hydrolysis of AAm, especially at high temperatures. Because of these two processes, initial shrinkage should be followed by reswelling, as is suggested from the data collected at 50 °C in Figure 5d.

## 3. Discussion

The present work represents a suite of observations regarding moderately substituted, sugar sensitive hydrogels based on p(AAM-*co*-MPBA) hydrogels. Some of our observations may depend on the relatively high (20 mol %) incorporation of MPBA, and may not be relevant to systems in which MPBA incorporation is reduced, and/or a basic comonomer is incorporated [16,37,45]. Such comonomers obviate the need for hydroxide ion to convert the PBA unit into its tetragonal, sugar/binding form, although pH may affect relative occupancy of :OH^−^ versus amine on the boron atom [37]. The strong pH effect in the present system, even in the absence of sugars, necessitates inclusion of the Donnan/Langmuir terms in the swelling equilibrium model, which other researchers have assumed to be negligible [16].

In Section 2.1, we demonstrated that the FRDL model, augmented to include fructose binding, provides an adequate description of binding equilibria over a wide range of pH values and fructose concentrations. In fitting this model, we assumed that the polymerizations were essentially complete, with full incorporation of the MPBA units. This allowed us to fix the parameters $\varphi_{0}$ and $\sigma_{0}$, along with $C_{salt}$, which was prescribed by our swelling buffer. Although we still needed to fit four parameters, we gained confidence in them by the following reasoning. First, we showed that the fitted value of the crosslink density parameter, $\rho_{0}$, was consistent with mechanical measurements made in the absence of sugar or in the presence of fructose. With this parameter in hand, the interaction parameter, χ, was set by the low pH swelling limit. The close correspondence of the predicted and experimental high swelling limits in Figure 2 provides further confidence in the FRDL model. Finally, the parameters *pK_a_* and *K_S_* were set, respectively, by the pH range over which swelling increases in the absence of added fructose, and the acid shifts in the swelling isotherm with addition of fructose at different concentrations. These observations account, likely, for the tight confidence intervals reported for the parameter estimates.

There has been disagreement as to the importance of glucose-mediated transient covalent crosslinks on swelling and mechanical behavior. Again, what may be most important is the degree of substitution, since the probability of forming such crosslinks increases with concentration of tetragonal MPBA units. The initial peak followed by relaxation to the plateau is also indicative of transient crosslinks which constantly break and reform. Alexeev et al. assumed that transient crosslinks, while affecting swelling, do not affect elasticity at equilibrium. Our observation of an increased plateau modulus with glucose at high pH, however, suggests that the transient crosslinks may contribute to the modulus. Some degree of increase in equilibrium shear modulus with added glucose in PBA-amine gels has also been reported by Horkay et al. [16], but these authors also claimed that glucose causes hydrogel shrinkage, in part, by altering the polymer/solvent interaction, i.e. the value of χ. The latter mechanism seems unlikely in our studies, since no effect of glucose concentration on swelling was observed at low pH. Our model also differs from that of Horkay et al. [16] in that we do not incorporate a ϕ-dependent χ parameter.

In Section 2.2, we characterized the decay of stress using a stretched-exponential, KWW functional form, accounting for a spectrum of relaxations corresponding to crosslink exchanges and topological rearrangements of chains occurring over diverse length scales. This characterization is purely phenomenological, and a future goal is to provide a more detailed molecular level description. Such descriptions have been attempted for solvent-free elastomers with dynamic exchangeable crosslinks [46,47,48], although these systems were not swollen, and did not contain permanent crosslinks (see [49] for an exception). For non-crosslinked systems, stress ultimately decays to zero following imposition of strain, and several models have been proposed with functional forms that are not KWW, but do display similar long decay tails [48,50,51,52,53,54]. These models account for the fact that newly formed crosslinks preserve “memory” of the present crosslink structure, and that stress is transferred to these crosslinks as other crosslinks break. Since motions of these polymers and their associated exchangeable crosslink sites are not constrained by the permanent crosslinks, the physical picture is somewhat different from that of our system.

In Section 2.3, we provided evidence that the p(AAM-*co*-MPBA) hydrogels are chemically unstable in pH 7.4 PBS at body temperature or higher, due to oxidative deborylation. Such instability may be an important drawback when considering long term implantation of devices with hydrogel components containing PBA moieties. Besides O_2_, H_2_O_2_ and other reactive oxygen species could play a degradative role. Colvin and Jiang [55] have recently shown, however, that oxidative deborylation can be retarded in vivo when a thin (few nanometers thick) layer of platinum is sputtered onto a hydrogel containing PBA.

## 4. Conclusions

To conclude, this paper has provided several contributions regarding the response of p(AAM-*co*-MPBA) hydrogels to pH, fructose, and glucose. The studies with fructose, which does not form crosslinks, enabled a characterization of FRDL parameters which can later be used to model swelling equilibria in the presence of glucose. The mechanical relaxation phenomena observed with glucose are intrinsically interesting, and may require new modeling approaches to explain our observations. Finally, the swelling stability studies provide a background for the kind of investigations that will be needed in order to show the viability of PBA-based hydrogels for long-term in vivo applications.

## 5. Materials and Methods

### 5.1. Synthesis of MPBA

MPBA was synthesized from 3-aminophenylboronic acid hemisulfate (PBA^+^) by modification of procedure due to Shiino et al. [32]. Following dissolution of 10 g of PBA^+^ in 160 mL distilled water, chilled to 0 °C in an ice bath, pH of the solution was adjusted to 5.0 by adding NaOH. Next, 12.34 g of 64.5 mmol 1-ethyl-3-(3-dimethylaminopropyl) carbodiimide (EDC) was added, with continuous stirring under argon for 15 min. An equimolar solution (with respect to PBA^+^) of methacrylic acid (MAA), dissolved in 50 mL of distilled water and with pH adjusted to 5.0, was then added dropwise to the PBA^+^/EDAC solution, still under argon. Following a 90 min argon purge at 0 °C, the mixture was removed from the ice bath, and stirred overnight at room temperature. MPBA was then extracted with methyl *t*-butyl ether (MTBE) three times and roto-evaporated. The white paste that resulted was re-dissolved in distilled water (380 mL) with continuous stirring at 70 °C. Activated charcoal (500 mg) was added, and the suspension was stirred for 1 min at 70 °C. Removing the charcoal using a filter funnel and leaving the filtered solution overnight released a white crystalline MPBA powder. Molecular structure was verified by ^1^H-NMR.

### 5.2. p(AAm-co-MPBA) Hydrogels

Copolymer hydrogels containing MPBA and AAm (p(AAm-*co*-MPBA)) were synthesized by redox polymerization in an alkaline aqueous solution containing 80 mol % AAm and 20 mol % MPBA. The two comonomers were dissolved in 1 mL water containing 1 N NaOH, 80 µL of *N*,*N*,*N*′,*N*′-tetramethyl ethylenediamine (TEMED: accelerator) and 100 µL of 20 mg/mL methylenebisacrylamide (Bis: crosslinker). To this mixture, 0.2 mL of a 10 mg/mL aqueous solution of ammonium persulfate (APS: initiator) was added. The resulting pre-gel solution was introduced into silanized glass capillaries (I.D. = 1.172 mm) and polymerized at 4 °C for 12 h. Hydrogels were separated from the capillaries using acetone, cut into short cylinders, washed in distilled water, and equilibrated in pH 7.4 PBS at room temperature. In an alternate geometry, identically composed pre-gel solutions were poured into acrylic molds of diameter 8 mm and thickness 0.8 mm. Following polymerization, the disk-shaped hydrogels were removed, washed in distilled water, and equilibrated in pH 7.4 PBS at room temperature. Control cylindrical homopolymer (pAAm) hydrogels were synthesized as above, but with the MPBA replaced by the same mass of AAm.

### 5.3. Swelling Studies

Swelling equilibrium data, from hydrogels synthesized according to the methods just described, are taken from Ref [37]; we recapitulate here the methods of measurement. Swelling equilibria were measured at room temperature over pH values ranging from 4 to 10, and at specified fructose concentrations (0, 0.5, 2, 7, and 20 mM). Cylindrical hydrogels were immersed in 0.01M pH buffers, and adjusted to 0.155 M ionic strength with NaCl. Buffers were acetic acid (*pK_a_* = 4.76) from pH 4 to 5.5, sodium phosphate monobasic (*pK_a_* = 7.2) from pH 6 to pH 8, and ethanolamine (*pK_a_* = 9.5) from pH 8.5 to 10. The swelling medium for each sample was replaced daily. At the end of the swelling period, the final pH of the swelling medium was determined for each condition, and equilibrium diameter changes of the hydrogel cylinders were measured under an optical microscope with crosshairs, calibrated against a grid. Two days was sufficient for the cylindrical hydrogels to reach swelling equilibrium. All swelling measurements were carried out in duplicate, at room temperature.

### 5.4. Mechanical Measurements

Disk shaped hydrogels were equilibrated in PBS solutions containing fructose or glucose at 9 mM, or no sugar, at pH 7.4 or 10.0 at room temperature. Each disk was mounted between parallel plates of a rheometer (ARES, Rheometric Scientific, Inc., Piscataway, NJ, USA), with the top and bottom plates covered with thin acrylic sheeting to provide a low friction surface. The hydrogel was compressed from 100 to 90% thickness at constant velocity over selected durations of 1–100 s, held at fixed strain for 100 s or 5 min, and then decompressed. Compression force F(t) was continuously monitored during the compress–hold–decompress process. For analysis, force was converted to engineering stress, $F(t)/A_{i}$, where $A_{i}$ was the initial surface area of the hydrogel disk in its equilibrated state, exposed to the top plate. Single disks were used for each condition.

### 5.5. Thermal Degradation

Four thermal environments were prepared: a water bath at room temperature, an Eppendorf thermomixer 5436 at 37 °C, an incubator at 50 °C, and a heating block at 65 °C. Cylindrical hydrogels were placed into vials containing phosphate buffered saline (PBS: pH 7.4, ionic strength 155 mM) with either 0 or 9 mM fructose, and incubated for up to 50 days. At various times, the hydrogels were removed, and their diameters measured, as described above, and placed in vials containing fresh solution. All measurements were performed in duplicate.

## Figures and Tables

**Table:** 

Fixed Parameters	Fitted Parameters: 95% CI
$\sigma_{0}$ = 0.553 mol/cm^3^	χ = 0.61 ± 0.01
$\varphi_{0}$ = 0.242	$\rho_{0}$ = 7.26 ± 1.29 mmol/cm^3^
*C_salt_* = 0.155 mol/L	*pK_a_* = 8.20 ± 0.10
	*K_S_* = 0.09 mmol/L

**Table 1:** Measured plateau engineering stresses, $F_{\infty }/A_{i}$, and active chain densities, $\rho_{0}$, calculated using Equation (5) using data from Figure 3. Compression ratio is α = 0.9.

pH		$F_{\infty }/A_{0}$ (Pa)	*d/d*_0_	Calculated $\rho_{0}$ (mM)
**pH 10**	**0 mM**	3012	2.05	7.45
	**F9 mM**	2460	2.02	5.99
	**G9 mM**	5042	1.54	9.35
**pH 7.4**	**0 mM**	5131	1.13	6.96
	**F9 mM**	3200	1.86	7.19
	**G9 mM**	5031	1.26	7.62

**Table 2:** Fitted Kohlrausch–Williams–Watts (KWW) time and stretch constants stress relaxations recorded at plateau strain with hydrogels exposed to 9 mM glucose at pH 10, with different compression times.

$T_{comp}$(s)	τ (95% CI) (s)	β (95% CI) (Dimensionless)
1	3.75 ± 0.13	0.495 ± 0.011
10	3.68 ± 0.21	0.549 ± 0.013
100	15.49 ± 0.26	0.753 ± 0.016
